# Cone-Beam Computed Tomography as a Prediction Tool for Osteoporosis in Postmenopausal Women: A Systematic Literature Review

**DOI:** 10.3390/diagnostics13061027

**Published:** 2023-03-08

**Authors:** Abulfaz Isayev, Nigiar Velieva, Luljeta Isedisha, Zhala Isayeva, Kıvanç Kamburoğlu, Fatih Kuyumcu

**Affiliations:** 1 School of Dental Medicine, Boston University, Boston, MA 02118, USA; 2 Russian Medical Academy of Continuous Professional Education, Moscow 125993, Russia; 3 Department of Dentomaxillofacial Radiology, Faculty of Dentistry, Ankara University, Ankara 06560, Turkey; 4 Buyukcekmece Agiz ve Dis Sagligi Merkezi, Istanbul 34500, Turkey

**Keywords:** CBCT, dual energy X-ray absorptiometry, postmenopausal, bone mineral density, osteoporosis

## Abstract

This literature review was conducted to analyze the capability of cone-beam computed tomography (CBCT) to accurately identify low bone mass density in women. A systematic search of MEDLINE, Embase, Scopus, Google Scholar, the Cochrane Library, and Science Direct was performed to identify relevant articles, and the Cochrane risk of bias criterion was used to determine the methodological quality of the included studies. All ten included studies assessed primary research on the capacity of CBCT to accurately diagnose insufficient bone mineral density. All relevant data were extracted, and the results were summarized narratively. The results indicated that the CBCT has good sensitivity and specificity and high accuracy in predicting osteoporosis. Four of the included studies measured qualitative values, while the others concentrated on quantitative values and found lower values in osteoporosis patients compared to those of osteopenic and healthy patients. All the studies compared CBCT grayscale values with dual energy X-ray absorptiometry scores, which strengthened our confidence in the accuracy of CBCT’s diagnostic capability. CBCT is considered a feasible predictive tool for detecting patients who are at risk of osteoporosis, although further research is needed to confirm the evidence and enhance its common use among health care professionals.

## 1. Introduction

Osteoporosis, a silent skeletal disease, is characterized by low bone mass and deterioration of the bone structure, which compromises bone strength and increases the risk of bone fractures in any area of the skeleton, excluding the face [1]. It occurs as a result of the aging process; however, there is a progressive and higher rate of bone loss in postmenopausal women. Following this period, bone loss declines at a more gradual rate [1].

The diagnosis of osteoporosis is confirmed by measuring a patient’s bone mineral density (BMD), which allows the delivery of preventive and therapeutic intervention if necessary. BMD is defined through the values of the T- and Z-scores. The mean value of standard deviations (SD) in young, healthy individuals is a reference for the T-score, while the mean values of SD expected for healthy individuals within the same age and sex group determine the Z-score. A T-value of 2.5 SD or more below the mean for a young female adult is an indication of osteoporosis, whereas a T-score between −1 and −2.5 SD is used to define osteopenia [2]. Although Z-scores are not routinely used to diagnose osteoporosis, low Z-scores can indicate osteoporosis, and the patient should be evaluated for metabolic bone diseases or secondary causes of osteoporosis [3].

Osteoporosis exerts a significant social and economic impact on the global population due to high treatment costs and a humanistic burden [4]. According to international guidelines, all women over 65 years of age should undergo bone densitometry, as should younger postmenopausal women with associated risk factors [5], such as amenorrhea, low estrogen levels, hyperthyroidism, chronic rheumatoid arthritis, and chronic hepatitis C [6].

The silent nature of this condition contributes to the difficulty of its detection and diagnosis. Hence, all medical professionals should participate in identifying osteoporotic patients and refer them to specialists for a timely diagnosis and suitable treatment [7]. Early detection may be especially crucial in dentistry as it has the potential to prevent higher failure rates of dental implants. Dentists are commonly consulted by the vast majority of the population, and dental radiographs are widely used to diagnose teeth and jaw conditions in the adult population. These radiographs can be used as a screening tool for osteoporosis [8].

Dual energy X-ray absorptiometry (DXA) is recognized as the gold standard method for identifying those at risk for low BMD. DXA is an accurate and non-invasive method; however, high radiation doses and costs make the application of this tool unfeasible for routine examinations [9]. Although the proximal femur and lumbar spine are primarily used to assess BMD by DXA, researchers have performed studies on panoramic images and developed different qualitative and quantitative radiomorphometric indices to predict low BMD with less cost burden and radioactive exposure to patients. Studies have shown that the mandibular cortical index [10] and the mental index [11] are the most effective methods for predicting osteoporosis on two-dimensional (2D) images [12].

Cone-beam computed tomography (CBCT) has been widely used since its introduction in dentistry in 1998 [13] and has become an essential tool in modern dental practices. According to a 2019 survey by the American Dental Association, 47% of general dentists reported having a CBCT machine in their practice, and the number is likely higher for specialty practices such as endodontics, oral and maxillofacial surgery, and orthodontics. In Europe, a study conducted in 2017 found that 85% of respondents reported having access to CBCT technology in their dental practice, with the highest usage reported in Scandinavian countries and the lowest in Eastern Europe. Overall, the use of CBCT technology in dental practices is growing, and it is becoming an increasingly essential tool for diagnosing and treating dental conditions. CBCT provides three-dimensional images of the teeth, jaw, and surrounding structures, allowing dentists to more accurately diagnose and plan treatments. It has several applications in dentistry, including implant placement, endodontic therapy, orthodontic treatment planning, and evaluating the extent of oral pathology. CBCT enables a submillimetric accuracy assessment of bone quantity [14] and offers 2D and three-dimensional (3D) images at lower radiation doses and costs than medical computed tomography, with improved image sharpness and minimal distortion [15]. Previous studies have provided some evidence of CBCT’s capacity to detect low BMD and suggested the use of CBCT as an early screening tool. Moreover, a previous systematic review [15] compiled evidence of the CBCT’s capacity to detect BMD. However, low BMD is primarily a health issue for women, and the previous review included evidence for both males and females. Therefore, this systematic review was conducted to identify evidence demonstrating CBCT’s capacity to diagnose low BMD in the female population.

## 2. Materials and Methods

The protocol for this systematic review was registered at PROSPERO (the International Prospective Register of Systematic Reviews) under the number CRD209839. The study complies with the Preferred Reporting Items for Systematic Reviews and Meta-Analyses (PRISMA) reporting guidelines for a systematic literature review [16]. We applied a rigorous and transparent methodology to reduce bias in the selection of relevant studies. The purpose of this study was to identify the diagnostic accuracy of CBCT to predict osteoporosis in the postmenopausal female population.

### 2.1. Eligibility Criteria

Comparative diagnostic studies assessing the capacity of the CBCT scan to identify low BMD in comparison to that of the gold standard DXA were included in this study. Studies assessing other imaging technologies, mixed population (premenopausal and postmenopausal) technologies, non-comparative studies, and studies comparing the diagnostic capacity of CBCT with technologies other than DXA were excluded. Only those studies conducted on adult female participants and published in the English language were included in this systematic review. Randomized controlled trials, non-randomized studies, cohort studies, case-control studies, cross-sectional studies of systematic literature reviews, and network meta-analyses were included in this review. Letters to the editors, narrative reviews, editorials, expert opinions, case studies, and book chapters were excluded. A detailed eligibility criterion is reported below in Table 1.

### 2.2. Data Sources and Search Strategy

A search of the MEDLINE, Embase, Scopus, Google Scholar, Cochrane Library, and Science Direct databases was conducted for this review. The search strategy used for the Embase and MEDLINE databases was generated from search terms related to CBCT, DXA, and osteoporosis. Filters were applied to limit the studies only to those on humans and adults that were published in the English language. The search was not limited by any geographical location. References from the compatible studies were also searched to identify similar studies. Final data were downloaded and uploaded into the reference management software “Zotero” [17].

### 2.3. Study Selection

Abstracts were assessed against the eligibility criteria shown in Table 1. The studies were screened using the abstract screening software “Rayyan” [18]. All articles underwent a two-stage selection process. During the first stage, two authors (A.I. and N.V.) independently reviewed the articles’ titles and abstracts, and the full texts were obtained for all eligible selections. The full texts were then screened using the same inclusion criteria as the abstract screening while identifying the methods used in the studies. In the second stage, two authors assessed the selected articles together and excluded those that did not meet the inclusion criteria. Two researchers independently conducted full-text screening and resolved disagreements through discussion and consultation with a third reviewer (K.K.). Three authors conducted the final evaluation, and any discrepancies were resolved through discussion and consensus.

### 2.4. Data Extraction

The relevant data were extracted into a previously agreed-upon Microsoft Excel template. The data extracted for each eligible study included the following:

- Study characteristics: study name, authors, title of the study, objectives of the study, study design, year of publication, study setting, sampling design, sample size, and country of origin;
- Patients’ characteristics: study population (diagnosis), age, co-morbidities, previous fractures, and patients’ classification;
- Intervention (CBCT) characteristics: unit, version, field of view, voxel size, and viewer software;
- Comparator (DXA) characteristics: name, reference, and characteristics;
- Outcomes: sensitivity, specificity, accuracy, CBCT grayscale values, DXA T-score, and Z-score.

### 2.5. Quality Appraisal

All included studies were assessed using a revised diagnostic accuracy quality assessment tool, the Quality Assessment Tool for Diagnostic Accuracy Studies-2 (QUADAS-2) [19].

### 2.6. Synthesis of Findings

A narrative synthesis was performed to synthesize the findings of the included studies as the studies were heterogeneous due to the variations in the index test devices, areas assessed, and analytical approaches.

A preliminary synthesis was conducted in the form of a thematic analysis that involved study characteristics and results in tabular form. The results were then reviewed and structured into themes. Included studies were summarized narratively in each theme. The themes were based on the sampling design of each study and the risk of bias criteria. Within each theme, the outcomes were summarized in groups as our outcome measures varied considerably among various studies. This framework comprised the study design (case-control and cohort) and the accuracy measures (sensitivity and specificity).

## 3. Results

**Figure 1:**
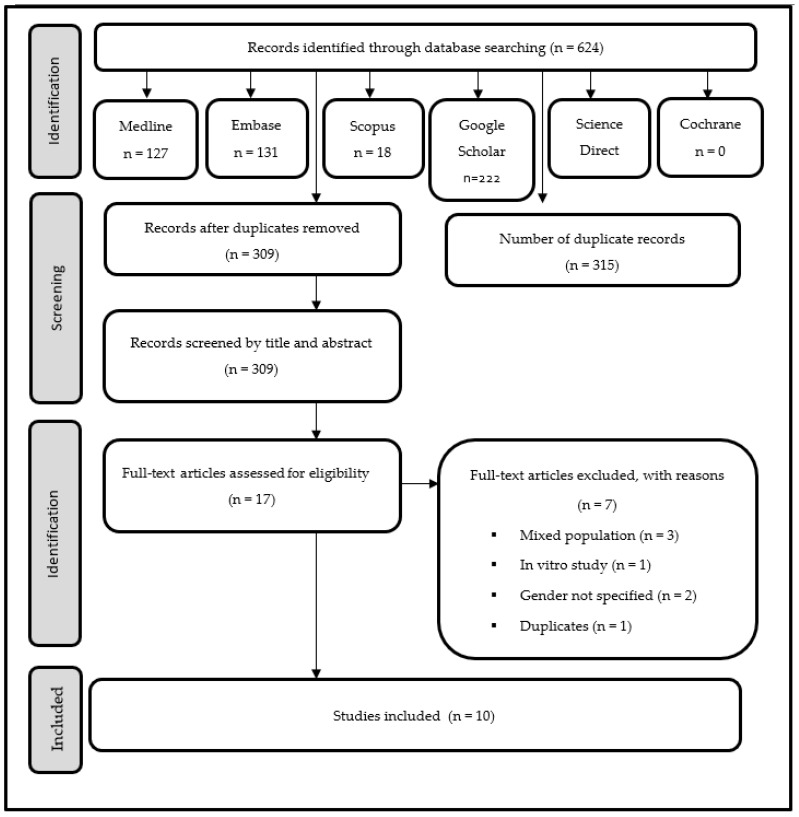
PRISMA flow diagram.

In the first phase of the selection, 624 references were identified, of which 315 were duplicates, leaving 309 unique references for the screening phase. The full texts of seventeen potential articles were retrieved for review. However, seven of these articles were excluded for not meeting the determined inclusion criteria. The remaining ten studies met the criteria for inclusion in this systematic review for final analysis (Figure 1).

### 3.1. Study Characteristics

All ten included studies investigated the capacity of dental CBCT imaging to accurately diagnose osteoporosis using observational cross-sectional methodologies. Three studies each were conducted in Syria [7,8,20] and Brazil [4,9,21], and the remaining four studies were conducted in Iran [22], Egypt [23], Korea [24], and the United States [25].

A total of 510 participants were enrolled across the ten studies. Case-control was the most common method of sampling and was used in six of the ten studies [4,7,8,20,23,24] while cohort sampling was used in the remaining four studies [9,21,22,25]. A summary of the included studies is reported in Table 2.

### 3.2. Participants’ Characteristics

Among the female participants, three of the ten studies enrolled menopausal and postmenopausal women [7,8,20], while seven of the ten studies enrolled postmenopausal women only [4,9,21,22,23,24,25]. Participants’ ages ranged from 46 to 83 years across the studies.

### 3.3. Intervention (CBCT) Characteristics

The studies demonstrated extensive variation in the characteristics of CBCT devices used to assess low BMD. Three studies used WhiteFox^®^ version 3 [7,8,20], two used the KODAK 9000 C 3Dsystem [9,21], and the remaining five studies used different devices. The field of view, voxel size, and viewer software varied across the included studies. The characteristics of CBCT devices are reported in Table 2.

### 3.4. Quality Assessment

**Figure 2:**
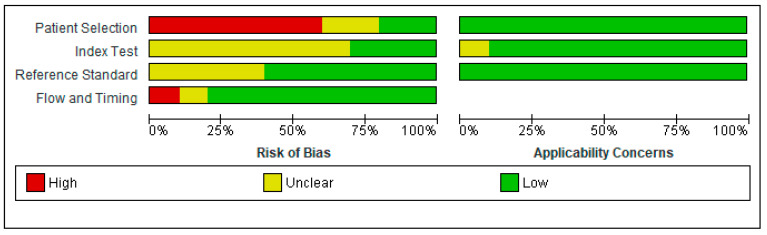
Risk of bias and applicability concerns graph.

**Figure 3:**
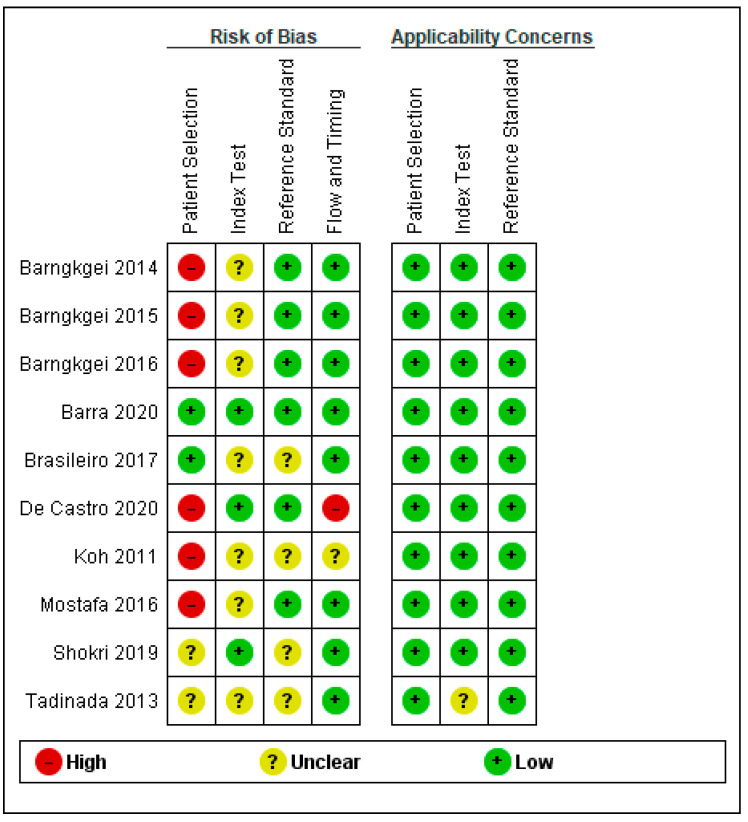
Summary of risk of bias and applicability concerns [4,7,8,9,20,21,22,23,24,25].

Quality assessment and applicability concerns for each of the included articles are outlined in Figure 2 and Figure 3 and represent a review of the authors’ judgments regarding each section across the included studies. Overall, there were large variations among studies in case and control classifications, CBCT devices, and viewer software. Small sample sizes and insufficient data ruled out the performance of a meta-analysis. Only one study fulfilled all the methodological quality criteria [9].

#### 3.4.1. Patient Selection

Patient selection posed a high risk of bias (ROB) in six of the ten studies that utilized case-control sampling. Two studies were considered to have a low ROB because of the use of cohort sampling techniques and random sampling strategies. The ROB was unclear for two [22,25] of ten studies. Shokri et al. (2019) [22] avoided a case-control design for patient selection but did not report the sampling method, whereas Tadinada, Raisz, and Lurie (2013) [25] did not present sufficient information to assess selection bias. Patients and settings did coincide with the review question; hence, the applicability concern was low for all ten studies.

#### 3.4.2. Index Test

The ROB for the index text was unclear in seven of the ten studies, primarily due to a lack of information about masking for conducting and interpreting the index test results. Moreover, the authors in these studies provided no information on the use and pre-specification of thresholds. However, the remaining three studies had low ROB [4,9,22]. Index test applicability concerns were low in all studies except one, which had an unclear applicability concern due to a lack of information.

#### 3.4.3. Reference Test

Regarding the reference test, the ROB was low in six of the ten studies. However, the remaining four studies had an unclear ROB due to the lack of data blinding; thus, it was not clear whether the findings were evaluated without knowing the results of the study. Reference test applicability concerns were low in all studies except one, which had an unclarified applicability concern due to a lack of information.

#### 3.4.4. Flow and Timing

The ROB for flow and timing was high in the study conducted by de Castro et al. (2020) [4]. Although the time interval between the index and reference tests was not a matter of concern, the authors excluded osteopenia patients, which raised a concern for a false increase in sensitivity; hence, we considered it to have a high ROB. The ROB was unclear in the study by Koh and Kim (2011) [24], as the authors did not report the timing between two tests. All the other included studies had a low ROB for flow and timing.

### 3.5. Outcomes

The reporting of outcome variables varied across the included studies. Six of the ten studies reported sensitivity and specificity [4,7,8,9,20,22], while accuracy was reported in half of the included studies [3,5,7,8,18]. Four of the included studies measured qualitative radiomorphometric indices and linear measurements on the CBCT scans [9,21,23,24]. The study included three publications by Barngkgei et al. (2014, 2015, 2016), who all conducted research on a single group of postmenopausal women [7,8,20].

Brasileiro et al. [21] performed measurements on CBCT-derived cross-sectional images to determine the mean values of the computed tomography mandibular index, the computed tomography index (inferior), and the computed tomography index (superior). The authors concluded that the mean values of all indices were significantly lower in the osteoporosis group compared to those of osteopenic and healthy patients (*p* < 0.02) [21].

Mostafa, Arnout, and Abo El-Fotouh [23] assessed the capability of using box-counting fractal dimension (FD) and mandibular CBCT radiomorphometric indices to detect osteoporosis in 25 postmenopausal women and compared their results to those of a control group that included 25 healthy females. The authors found significant differences in the CT mental index, CT cortical index scores, and CT mandibular index between the cases and control groups. The osteoporotic group showed lower mean values compared to those of the healthy group. However, the FD values of the control group were not significantly higher than those in the osteoporotic group. The authors suggested that CBCT is a useful secondary diagnostic tool to implement in patients at risk of insufficient bone density [23].

Koh and Kim [24] studied the possible use of radiomorphometric indices derived from CBCT images for the diagnosis of osteoporosis in a sample of 21 postmenopausal osteoporotic women and 21 postmenopausal healthy women. The authors found that there were statistically meaningful differences between the two groups in the values of the superior, inferior, and cortical computed tomography indexes, whereas no statistically significant difference was observed in the values of the computed tomography mental index between the two groups [24].

Barra et al. [9] evaluated four new radiomorphometric indices: symphysis, anterior, molar, and posterior. The authors found that there was no statistically significant difference in the mean values of the symphysis and anterior indexes between the normal, osteopenia, and osteoporosis groups. However, the mean molar index showed noticeably lower values in osteopenia (*p* < 0.001) and osteoporosis (*p* = 0.001) as compared to those of healthy individuals. The mean posterior index was markedly lower in patients with osteoporosis than in those of healthy patients (*p* = 0.008), while no significant difference in the mean posterior index was found between the osteopenia and healthy groups (*p* = 0.031). These findings demonstrate that the molar and posterior indices may become useful tools in dental CBCT images of the mandible for the evaluation of bone density and the diagnosis of osteoporosis in postmenopausal women [9].

Barngkgei et al. (2014, 2015) studied lumbar spine and femoral neck T-scores and their correlation with radiographic density (RD) derived from dental CBCT for the evaluation of osteoporosis in postmenopausal females [7,8]. The authors demonstrated that RD values derived from the CBCT of the mandibular slice that passes through the lower borders of the mental foramina show high accuracy in predicting osteoporosis and correlate with the femoral neck and lumbar T-scores.

Barngkgei, Joury, and Jawad [8] also assessed RD from the cervical vertebrae. The RD values derived from CBCT of the left part of the atlas (C1) and the dens (odontoid process of the second cervical vertebra) showed the strongest correlation coefficients (r = 0.6 and 0.7; *p* < 0.001) and the highest sensitivity (70% and 76.9%), specificity (92.9% and 92%), and accuracy (86.4% and 90.8%) in predicting osteoporosis in the femoral neck and the lumbar vertebrae, respectively [8]. The dens and the trabecular bone structure of the jawbones of osteoporotic and non-osteoporotic women were assessed by using CBCT in a separate study. Barngkgei et al. [20] found minor differences in the jawbone-derived measures between the osteoporotic and non-osteoporotic groups. The correlation of the jawbone-derived measures with the femoral neck and lumber vertebrae T-scores (between osteoporotic and non-osteoporotic groups) was ≤0.4 (*p* > 0.05). However, the correlation between the dens-derived measures and the femoral neck or lumber neck was from 0.34 to 0.38 (*p* = 0.02–0.036) and from 0.48 to 0.61 (*p* ≤ 0.003), respectively [20].

de Castro et al. [4] used cross-sectional and panoramic reconstructed images to evaluate mandibular cortical width and cortical quality. The authors found significantly lower mandibular cortical width values in osteoporotic women as compared to those of women with normal BMD according to DXA measurements at all three bone sites: Lumbar spine, femoral neck, and total hip [4]. A new 3D morphometric index on panoramic reconstructed images derived from CBCT has been found to be helpful for assessing the osteoporosis status of postmenopausal women.

Shokri et al. [22] assessed the correlation between BMD determined by CBCT gray values and BMD determined by DXA. The authors found that 61 asymptomatic patients (47.5%) had an abnormal BMD based on the T-scores of the femoral neck, while 55.7% of the patients had an abnormal BMD based on the T-scores of the lumbar spine. The correlations between the T-scores and the gray values of the maxillary incisor and tuberosity areas were significant [22].

Tadinada, Raisz, and Lurie [25] investigated the accuracy of pixel intensity values and width of the mandibular inferior cortical border measured on images derived from a CBCT in comparison to T-scores on 32 postmenopausal women. The authors found a strong correlation between the right and left mental indices, but no statistically significant correlation was found between the T-scores of the hip and lumbar vertebra and the mental index values on the right or left side. A strong correlation was found between the thickness of the mandibular cortical border, pixel intensity values, and age. There was no correlation between pixel intensity values and T-scores.

## 4. Discussion

This systematic review evaluated the available literature and compiled evidence regarding the capacity of a CBCT scan to accurately detect low BMD. All ten studies included in this systematic review provided evidence on the CBCT’s capability to accurately diagnose bone density and quality. All studies compared CBCT grayscale values with DXA scores, which strengthened our confidence in the accuracy of CBCT’s diagnostic capacity. Furthermore, six of the ten studies reported sensitivity and specificity, and four studies used radiomorphometric indices to successfully differentiate osteoporotic and non-osteoporotic individuals [9,21,23,24].

Bone density is the main factor influencing bone strength. Therefore, osteoporosis-related traumatic fractures occur in patients with compromised bone strength where bone microstructure changes and bone density is reduced [23]. Although osteoporotic fractures can affect any area of the skeleton except the face, fractures involving the hip have the highest associated mortality and morbidity and may cause death in 10–20% of cases within the first year. The survivors mostly suffer from disabilities that significantly affect their quality of life [7].

The findings of this systematic review are in line with those of a previous review reported by Guerra et al. [15]. They considered CBCT to be a promising tool for identifying low BMD in patients but emphasized the need for standardized studies with larger sample sizes. Their main concern was the heterogeneity of their available data. Although more articles have been reported since that review, the lack of homogeneous studies remains a concern. The main difference between the previous review and the present study is the included population criteria. Analyzing only the female population limits the picture of the whole population. Thus, studies reporting only male BMD and osteoporosis status are needed.

In our systematic review, we identified several studies that investigated the use of radiomorphometric indices derived from CBCT images for assessing osteoporotic changes in the jawbone. Güngör et al. (2016) [26] also studied radiomorphometric indices using CBCT images and concluded that osteoporotic changes could be accurately assessed through CBCT scans. However, we did not include this study in our systematic review as the authors did not report participants’ sex.

In a mixed population study conducted in Saudi Arabia [27]. T-scores and grayscale values were obtained from all 81 participants. The authors concluded that the CBCT grayscale values of BMD at different jawbone regions significantly correlated with the DXA T-values in osteoporotic patients. These findings are similar to those of other studies included in our review that have found significant correlations between CBCT radiomorphometric indices and DXA measurements of BMD in osteoporotic patients.

There are limitations in this review that should be considered. Although the methodological quality of all included studies was carefully considered to mitigate potential bias, many of the included studies failed to pay due diligence to patient sampling and selection. Moreover, small sample sizes, convenience sampling, and case-control study designs played a major role in introducing bias in many of the included studies. Although case-control studies are less expensive and easier to perform, they may lead to a 2- or 3-fold increase in diagnostic accuracy compared to prospective cohort studies [28].

Ideally, an index test should be performed before the gold standard test. However, in most of the included studies, DXA was performed before CBCT. In fact, participants were selected based on the DXA results. Many of the included studies failed to report test interpretation blinding, which might have induced bias in reporting. Likewise, some of the included studies excluded patients with osteopenia, which could falsely increase the sensitivity of the test.

Furthermore, there were large variations among studies in cases and control classifications, CBCT devices, and viewer software. Small sample sizes and insufficient data ruled out the ability to perform a meta-analysis. Only one study fulfilled all the methodological quality criteria [9].

It is important to note that the language limitation of this systematic review may have resulted in the exclusion of relevant studies published in languages other than English. Therefore, the findings presented in this review may not be representative of the entire body of literature on the topic. Future research should consider including studies in other languages to provide a more comprehensive understanding of the subject matter.

While some articles included in this review contain quantitative metrics, the heterogeneity of the data was considered a major restriction in performing a meta-analysis. The designs of the included studies were either unclear or not stated on a standard basis. In the future, high-quality studies with standardized CBCT devices and viewer software will be needed to ensure accuracy and reproducibility.

## 5. Conclusions

This systematic literature review provided cumulative evidence suggesting that CBCT is capable of accurately detecting low BMD. Extending the use of CBCT in dental practices to detect osteoporosis may help identify high-risk patients and in turn, reduce the social and economic burden associated with the disease.

## Figures and Tables

**Table 1:** Inclusion and exclusion criteria.

Category	Inclusion Criteria	Exclusion Criteria
Population	Adult female patients; Studies assessing the capacity of the dental CBCT scan for assessing low BMD in comparison to DXA.	Studies assessing other imaging technologies; Mixed population studies; Non-comparative studies; Studies comparing the diagnostic capacity of CBCT with technologies other than DXA.
Intervention	CBCT.	Studies with imaging modalities other than CBCT.
Comparator	DXA.	Studies with a comparator other than DXA; Non-comparative studies, i.e., studies without a DXA comparator.
Outcomes	Sensitivity; Specificity; Accuracy.	
Study design	Randomized controlled trials; Non-randomized studies; Cohort studies; Case-control studies; Cross-sectional studies.	Letters to the editor; Narrative reviews; Editorials; Expert opinions; Case studies; Book chapters.
Language	English language	Non-English language

CBCT: cone-beam computed tomography; DXA: dual energy X-ray absorptiometry.

**Table 2:** Summary of included studies.

Study Characteristics (Author, Year, Country of Origin)	Sample Characteristic Sample Size (n) Age (Mean ± SD) Years	Index Test (CBCT) Characteristics	Reference (DXA) Standards	Methodology	Conclusions
Barngkgei et al. [7] 2015 Syria	A total of 38 menopausal and postmenopausal women 57.9 ± 7.2.	WhiteFox^®^ version 3 Viewer software: WhiteFox Imaging^®^ Version 3	DXA Hologic Discovery QDR^®^	The lumber spine and femoral neck were analyzed. T-scores were calculated based on the DXA manufacturer’s guidelines. Radiographic density (RD) derived from CBCT images of the mandible and ramus was used to calculate gray values.	RD values of the body of the mandible on the CBCT-viewer program can be used to diagnose lumbar vertebrae and femoral neck osteoporosis.
Barngkgei et al. [8] 2015 Syria	A total of 38 menopausal and postmenopausal women 57.9 ± 7.2.	WhiteFox^®^ version 3 Viewer software: WhiteFox Imaging^®^ Version 3	DXA Hologic Discovery QDR^®^	The lumbar spine (L1–L4) and the femoral neck were analyzed. T-scores were calculated based on the DXA manufacturer’s guidelines. A coronal slice of the cervical vertebrae was selected, and the RD for the first and second vertebrae and the dens were calculated by using CBCT-viewer software (WhiteFox imaging).	CBCT-derived RD of cervical vertebrae can predict osteoporosis status using a CBCT-viewer program.
Barngkgei et al. [20] 2016 Syria	A total of 38 menopausal and postmenopausal women 57.9 ± 7.2.	WhiteFox^®^ version 3 Viewer software: WhiteFox Imaging^®^ Version 3	DXA Hologic Discovery QDR^®^	The lumbar spine (L1–L4) and the femoral neck were analyzed. T-scores were calculated based on the DXA manufacturer’s guidelines. Cuboids from various areas of the dens and jawbone were selected from each scan. Trabecular separation, trabecular thickness, bone volume fraction, connectivity density, and specific bone surface were measured.	Dens trabecular bone is affected in osteoporosis, as assessed by CBCT analysis.
Barra et al. [9], 2021 Brazil	A total of 48 postmenopausal women 61.4 ± 8.2.	KODAK 9000 C 3-D Viewer software: Implant Viewer program version 3.5	Hologic Discovery DXA System	Mandibular inferior cortical bone thickness was evaluated. Cross-sectional images at four sites were assessed and divided into osteoporosis, osteopenia, and normal groups. The lumbar spine (L1–L4) and proximal femur (neck and total) regions were examined.	M and P indices in the CBCT can identify low BMD in postmenopausal women.
Brasileiro et al. [21] 2017 Brazil	A total of 60 postmenopausal women. Normal: 55.60 Osteopenia: 55.20 Osteoporosis: 58.66	KODAK 9000C 3D^®^ Viewer software: Implant Viewer software^®^	Hologic Discovery DXA System^®^	Cross-sectional images generated to measure the CTMI, CTI (S), and CTI (I).	Quantitative CBCT indices may help dentists to screen for women with low spinal and femoral BMD so that they can refer postmenopausal women for bone densitometry.
de Castro et al. [4] 2020 Brazil	A total of 103 postmenopausal women: 52 normal and 51 osteoporotic. Normal: 64.8 ± 9.8 Osteoporosis: 63.9 ± 9.9	I-CAT Classic device. Viewer software: CBCT manufacturer software (Xoran 3.1.62)	DXA	Mandibular cortical width and cortical quality were evaluated on cross-sectional and panoramic reconstructed images. The selected lumbar spine (L1–L4) and hip DXA scans were performed.	The newly developed 3D MOI enables distinguishing women with osteoporosis from those with normal BMD with good sensitivity and specificity.
Koh and Kim [24] 2011 Korea	A total of 42 postmenopausal women: 21 osteoporotic and 21 healthy. Healthy: 60 ± 5.7 Osteoporotic: 66 ± 6.4	PSR-9000NTM Dental Viewer software: OnDemend3DTM	DXA scanner, Hologic Discovery QDR^®^, Hologic Inc.	Density of the femur and lumbar vertebrae by DXA. The axial, coronal, and sagittal samples were generated from the block images using OnDemend3DTM.	The computed tomography index (superior, inferior, or cortex) on the CBCT images can be used effectively in the diagnosis of osteoporotic women.
Mostafa et al. [23] 2016 Egypt	A total of 50 postmenopausal females Normal: 60.1 ± 3.7 Osteoporotic: 61.1 ± 4.9	Planmeca ProMax^®^ 3D Viewer software: Planmeca Romexis^®^	DXA	Mandibular CBCT radiomorphometric indices and fractal dimension analysis were measured.	CBCT radiomorphometric indices can be used as a secondary tool to assess populations at risk for osteoporosis.
Shokri et al. [22] 2019 Iran	A total of 61 postmenopausal women.	Scanora 3-D CBCT Viewer software: OnDemand 3D Dental software	DXA	Six regions in the jaws were chosen for assessment of BMD to identify the region of the jaw that had the highest correlation with the results of DXA. Locations included: between the mandibular incisor roots and the maxillary incisor roots; the left maxillary premolar roots and the left mandibular premolar roots; in the retromolar area of the left mandible; and in the left maxillary tuberosity. DXA: bone densitometry of the femoral neck and lumbar spine	The correlation between the CBCT gray values at different sites in the maxilla and DXA scores is strong. A gray value of less than 298 at the maxillary tuberosity can help identify patients with osteoporosis with an accuracy from 66% to 67% and suggests the need for DXA analysis.
Tadinada et al. [25] 2013 United States	A total of 32 postmenopausal women.	Six inch field of view CBCT exam of the facial skeleton	DXA	Inferior cortical border thickness and pixel intensity values over a one mm^2^ area in a standardized region inferior to the mental foramen measured on cross-sectional images from CBCT volumes were obtained.	The study did not show a correlation between the inferior alveolar cortex thickness, density, and DXA in this limited data set of patients.

## Data Availability

All data generated or analyzed during this study are included in this published article.
